# Effects of Prenatal Paracetamol Exposure on the Development of Asthma and Wheezing in Childhood: A Systematic Review and Meta-Analysis

**DOI:** 10.3390/jcm12051832

**Published:** 2023-02-24

**Authors:** Agnieszka Barańska, Wiesław Kanadys, Artur Wdowiak, Maria Malm, Agata Błaszczuk, Urszula Religioni, Anita Wdowiak-Filip, Małgorzata Polz-Dacewicz

**Affiliations:** 1 Department of Medical Informatics and Statistics with e-Health Laboratory, Medical University of Lublin, 20-954 Lublin, Poland; 2 Specialistic Medical Center Czechow, 20-848 Lublin, Poland; 3 Chair of Obstetrics and Gynecology, Medical University of Lublin, 20-081 Lublin, Poland; 4 Department of Virology with SARS Laboratory, Medical University of Lublin, 20-093 Lublin, Poland; 5 School of Public Health, Centre of Postgraduate Medical Education of Warsaw, 01-826 Warsaw, Poland; 6 Department of Cosmetology and Aesthetic Medicine, Medical University of Lublin, 20-093 Lublin, Poland

**Keywords:** paracetamol, asthma, wheeze, prenatal exposure, pregnancy, acetaminophen

## Abstract

The aim of the report was to evaluate whether in utero exposure to paracetamol is associated with risk towards developing respiratory disorders such as asthma and wheeze after birth. MEDLINE (PubMed), EMBASE and Cochrane Library databases were searched for articles published in English to December 2021. The study involved 330,550 women. We then calculated the summary risk estimates and 95% CIs and plotted forest plots using random effect models (DerSimonian–Laird method) and fixed effect models. We also performed a systematic review of the chosen articles and a meta-analysis of studies based on the guidelines outlined in the PRISMA statement. Accordingly, maternal exposure to paracetamol during pregnancy was associated with a significant increased risk of asthma: crude OR = 1.34, 95% CI: 1.22 to 1.48, *p* < 0.001; and significant increased risk of wheeze: crude OR = 1.31, 95% CI: 1.12 to 1.54, *p* < 0.002. Results of our study confirmed that maternal paracetamol use in pregnancy is associated with an enhanced risk of asthma and wheezing in their children. We believe paracetamol should be used with caution by pregnant women, and at the lowest effective dose, and for the shortest duration. Long-term use or the use of high doses should be limited to the indications recommended by a physician and with the mother-to-be under constant supervision.

## 1. Introduction

Paracetamol, also called “acetaminophen” or “N-acetyl-p-aminophenol” (APAP), is a mild-to-moderate antipyretic/analgesic drug widely used across the world, among a wide range of populations (from pregnant, pediatric and adult, to elderly people). At therapeutic doses, paracetamol is metabolized mainly by the formation of conjugates by glucuronidation and sulphation and is then excreted in the urine. Around 10% of all paracetamol is metabolized by cytochrome P450 (CYP) enzymes to form n-acetyl-p-benzoquinoneimine (NAPQI), which is subsequently conjugated with intracellular glutathione, and ultimately excreted as cysteine and mercapturic acid conjugates. Less than 5% is excreted unchanged [1,2,3].

Pregnancy is a special period in a woman’s life, characterized not only by metabolic and physiological changes in her organism, but also by the possibility of susceptibility to pathological conditions. This may have consequences for the fetus, as well as affect the further health of the woman [4]. Use of over-the-counter medications or drugs for acute/short-term illnesses and chronic/long-term disorders, as well as for temporary pain control, is common in pregnancy [5]. One of the more frequently employed drugs as an analgesic and antipyrotic during this period is paracetamol [6].

Cytochrome P450s metabolizes endogenous and exogenous substrates and is involved in metabolizing toxins and procarcinogens [7]. Therefore, paracetamol must be metabolized either to sulfate via sulfation or to N-acetyl-p-benzoquinone imine (NAPQI) via cytochrome P450s in early pregnancy [8,9]. Paracetamol freely crosses the placenta [10]; however, the fetus has a limited ability to metabolize paracetamol through glucoronidation. Detoxification of paracetamol may deplete stores of glutathione, leading to increased oxidative damage to the lung epithelium and, thus, contributing to wheezing or asthma [11,12,13].

The aim of this review and meta-analysis is to assess the relationship between prenatal paracetamol exposure and wheezing or asthma in children. 

## 2. Methods

We performed a systematic review of articles and a meta-analysis studies based on the guidelines outlined in the PRISMA statement [14].

### 2.1. Search Strategy

We considered all epidemiological studies that compared the risk of asthma or wheeze in childhood with prenatal paracetamol use. There were no limitations in searching for articles of interest in assessing the dependencies between prenatal paracetamol exposure and asthma or wheeze risk in childhood [15].

A thorough search was conducted in the electronic databases MEDLINE (PubMed), EMBASE and Cochrane to identify relevant research. Studies published up to December 2021 were included. The following search terms were used for all databases in various combinations: “asthma” or “wheeze” AND “paracetamol” or “acetaminophen” AND “prenatal” or “pregnancy”. Taking into account the possibility of not finding all the articles of interest to us during the database search, references lists of relevant articles were additionally analyzed. The search results were compared with previously published meta-analyses on this topic. All data were extracted by two investigators (A.B. and W.K.), and disagreements were resolved in discussion with a third investigator (A.W.).

### 2.2. Eligibility Criteria

Definitions that were adopted in our analysis include: “wheeze”—definition characterized by paroxysmal transient or persistent, symptoms affecting breathing, such as noisy breathing (“wheezing” or “whistling”), shortness of breath, or a troublesome cough affecting sleep or everyday activity; “asthma”—definition established by doctor’s diagnosis, clinical symptoms (shortness of breath, chest tightness or pain, cough, wheezing episodes) and/or use of asthma medication (note: certain differences in the definitions contained in some works made it difficult to qualify them to the finale definitions we have adopted).

The following inclusion criteria were established in the selection of studies: (i) trials that involve the comparison of women who used paracetamol during pregnancy with an observational group; (ii) studies evaluating the effect of prenatal paracetamol use on offspring, using wheeze or asthma as a primary outcomes; (iii) structure interview and clinical research; (iv) articles written in English; (v) data included in the articles were sufficient to calculate the odds ratio (OR) and 95% confidence interval (CI) and (vi) if there was an overlap in the cases included, only the latest and most comprehensive data were selected.

The exclusion criteria were as follows: (i) insufficient quantitative data (not possible to extract sufficient data for statistical calculations); (ii) duplicate reports; (iii) articles published in languages other than English and (iv) publications that were reviews, commentaries/letters, editorials, conference abstracts, cross-sectional studies. 

Full texts of potential articles were selected for evaluation on the basis of a review of the titles and/or abstracts of all identified studies. After analyzing the selected works, a decision was made to include or exclude them. Papers meeting these conditions were qualified for meta-analysis, data collection on clinical characteristics and for test statistics.

### 2.3. Data Abstraction

Extracted data included: age of children’s diagnosis and number of children with asthma or wheeze; number of women using paracetamol during pregnancy; trimester of pregnancy in which paracetamol use took place, and number of pregnant women in a particular trimester (if recorded).

### 2.4. Quality Assessment

The Newcastle-Ottawa Scale (NOS) was applied to assess the methodological quality of all the included studies [16]. The NOS included three categorical criteria with a maximum score of 9 points: (1) selection of the study group; (2) comparability of the groups; and (3) identification of the exposure for studies. The quality of each study was rated using the following scoring algorithms: ≥7 points were considered as “high”, 4 to 6 points were considered as “moderate”, and ≤3 point was considered as “low”.

### 2.5. Statistical Analysis

The distribution of cases, ORs and 95% CIs were separately identified based on the risk of childhood wheezing/asthma and prenatal exposure to paracetamol (ever or never) and use of paracetamol in each trimester (if available).

We calculated the summary risk estimates and 95% CIs and plotted forest plots using random-effects models (DerSimonian–Laird method) and fixed effect models for the association between prenatal paracetamol exposure and wheeze/asthma in childhood. The value of I^2^ statistics was adopted as a criterion—in the case of I^2^ < 50, we used a fixed effect model, and when I^2^ ≥ 50, a random effect model. The results indicated that the taking of paracetamol may have a high probability of increase in risk if OR was above 1, compared with non-use of paracetamol [17].

Heterogeneity among articles was estimated by engaging the I^2^ statistic and *p* values associated with Q statistics. Herein, I^2^ statistic indicates the percentage of total variability explained by heterogeneity, and values of ≤25%, 25%–75%, and ≥75% are arbitrarily considered as indicative of low, moderate, and high heterogeneity, respectively [18].

To explain the possible presence of publication bias, Begg’s test (a rank correlation method based on Kendall’s tau) and Egger’s test (a linear regression method) were applied [19,20]. We also checked for funnel plot symmetry. Here, in the absence of bias, the plots will resemble a symmetrical funnel, as the results of minor studies will scatter at the left side of the plot and the spread will narrow among the major studies on the right side of the plot [21]. Meta-analysis of summary statistics from individual studies was performed through Statistica 13.3 software (StatSoft Poland, Kraków, Poland), using the Medical Package program.

## 3. Results

**Figure 1:**
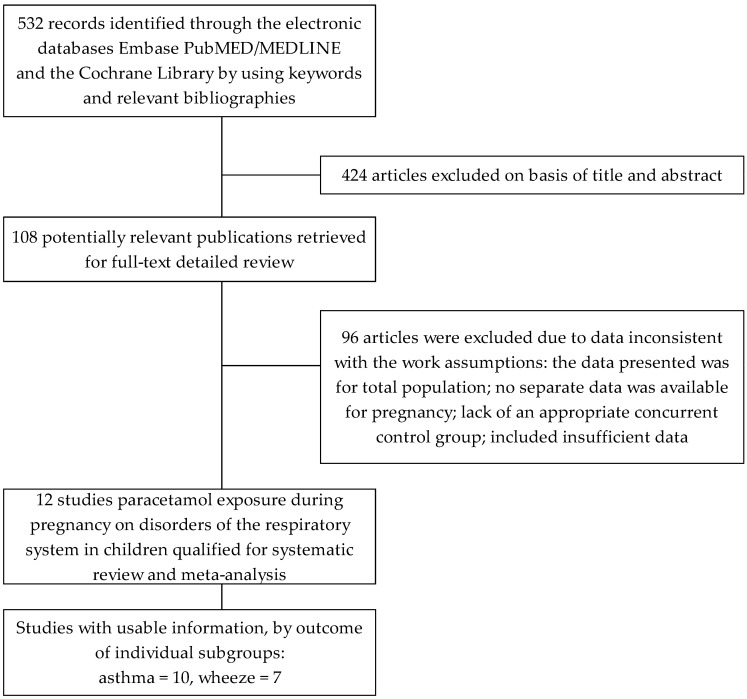
Flow diagram of literature search and research selection procedure.

As result of the search of electronic databases, 532 citations were identified. Titles and abstracts were checked in the initial selection phase, in which 424 items were excluded due to irrelevance. In the second phase, 108 articles with potentially significant studies were identified and submitted for full-text assessment. There were 96 papers which did not meet all the inclusion criteria, contained duplicate publications, and the required data were missing, amongst others. We identified twelve articles fulfilling the criteria for inclusion, in which the effect of paracetamol exposure during pregnancy on disorders of the respiratory system in children was analyzed [22,23,24,25,26,27,28,29,30,31,32,33]. The outcome of the search strategy is shown in Figure 1.

The studies involved 330,550 women and 44,502 women intake of paracetamol during pregnancy. Table 1 presents a tabular summary of the individual clinical–control studies discussed in this review. All studies included were in accordance with NOS scale and all studies were defined as high-quality. The average value was 8.03.

### 3.1. Sensitivity Analysis

In the study on the relationship between childhood asthma and paracetamol use (ever vs. never) during pregnancy and each trimester of pregnancy, sensitivity analysis showed that in the case of a total study and 3rd trimester, removing any of the studies would not significantly affect the result of the meta-analysis. However, in the case of the 1st trimester, deleting one of the studies: Andersen [27], Liu [25], Migliore [26] or Rebordosa [32] would change the result of the meta-analysis to be statistically insignificant. On the other hand, in the case of the 2nd trimester, the result of the meta-analysis would be statistically insignificant after excluding the study of Liu [25] or Rebordosa [32].

In the study on the relationship between childhood wheeze and paracetamol use (ever vs. never) during pregnancy and each trimester of pregnancy, sensitivity analysis for total study, 2nd trimester and 3rd trimester indicated that the results would not change significantly after excluding any of the studies. In turn, in the 1st trimester, the exclusion of the Liew study [22] would change the result of the meta-analysis to a statistically significant one.

### 3.2. Association between Paracetamol Exposure during Pregnancy and Asthma in Children

**Figure 2:**
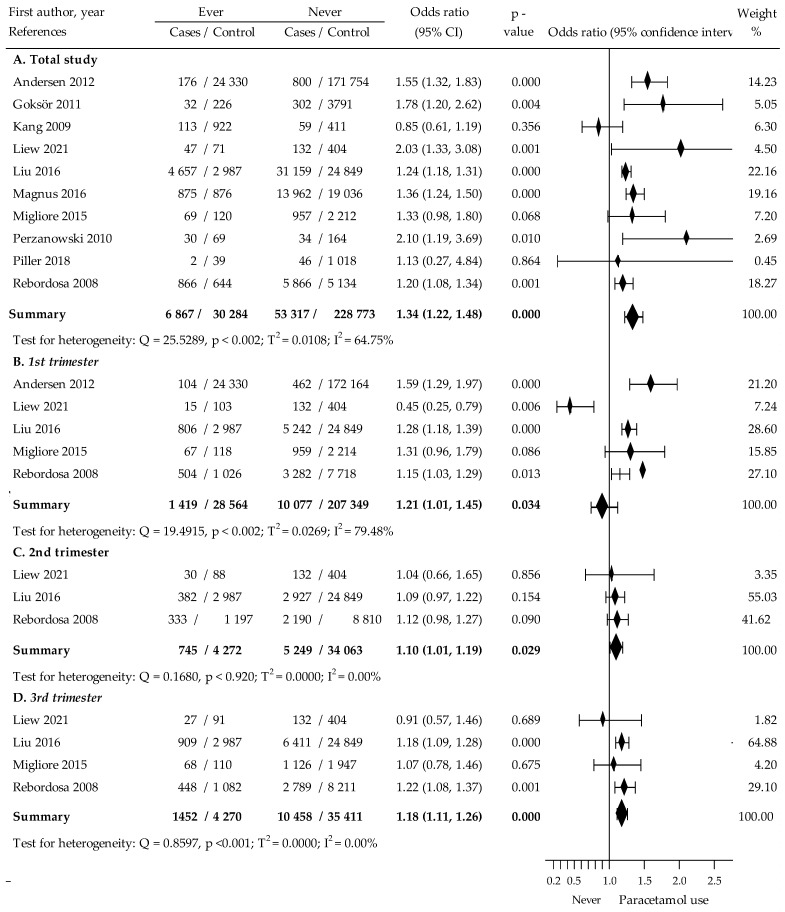
The crude relationship between childhood asthma and paracetamol use (ever vs. never) during pregnancy and each trimester of pregnancy [22,23,24,25,26,27,28,29,30,32].

The present meta-analysis was conducted on the basis of data from ten studies [22,23,24,25,26,27,28,29,30,32] assessing the effect of paracetamol exposure in pregnancy on the risk of occurrence of asthma in children. Paracetamol was taken at any time during the trimesters of pregnancy. The crude OR amounted to 1.34, 95% CI: 1.22 to 1.48, *p* <0.001, with moderate heterogeneity of I^2^ = 64.75% (Figure 2). The Begg and Mazumdar’s test for rank correlation did not indicate evidence of publication bias (Kendall’s tau = 0.142, z = 0.495, *p* < 0.622; similarly, Egger’s test: b0 = 0.966, 95% CI − 0.748 to 2.681, t = 1.299, *p* < 0.231).

Results of five studies [22,25,26,27,32] analyzing the relationship between of intake of paracetamol during first trimester and childhood asthma pointed to increased risk (crude OR = 1.21, 95% CI: 1.01 to 1.45, *p* < 0.035, I^2^ = 79.48%), (Figure 2). The Begg Mazumdar’s test and Egger’s test did not indicated evidence of publication bias (Kendall’s tau b = −1.000, z = −1.567, *p* < 0.118 and b0 = −1.288, 95% CI: −7.752 to 5.177, t = −0.634, *p* < 0.572, respectively). The major problem indicated by this analysis is the large heterogeneity of effect of paracetamol.

Further analysis involving three studies [22,25,32] also suggested that use of paracetamol during the second trimester of pregnancy was associated with increased childhood asthma risk (crude OR = 1.10, 95% CI: 1.01 to 1.19, *p* < 0.030, I^2^ = 0.00%), (Figure 2). Evidence of publication bias was not shown in the Begg and Mazumdar’s test (Kendall’s tau = 0.333, z = 0.522, *p* < 0.603); or in the Egger’s test (b0 = −0.237, 95% CI: −6.686 to 6.212, t = −0.468, *p* < 0.723).

In turn, meta-analysis based on the results of four studies [22,25,26,32] showed that paracetamol intake by women in the third trimester of pregnancy was associated with an enhanced risk of asthma in the child (crude OR = 1.18, 95% CI: 1.11 to 1.26, *p* < 0.001, I^2^ = 0.00%), (Figure 2). The Begg and Mazumdar’s test and Egger’s test did not indicate evidence of publication bias (Kendall’s tau = −0.667, z = −1.359, *p* = 0.174 and b0 = 0.966, 95% CI: −0.748 to 2.681, t = 1.299, *p* < 0.231, respectively).

### 3.3. Association between Paracetamol Exposure during Pregnancy and Wheezing in Children

**Figure 3:**
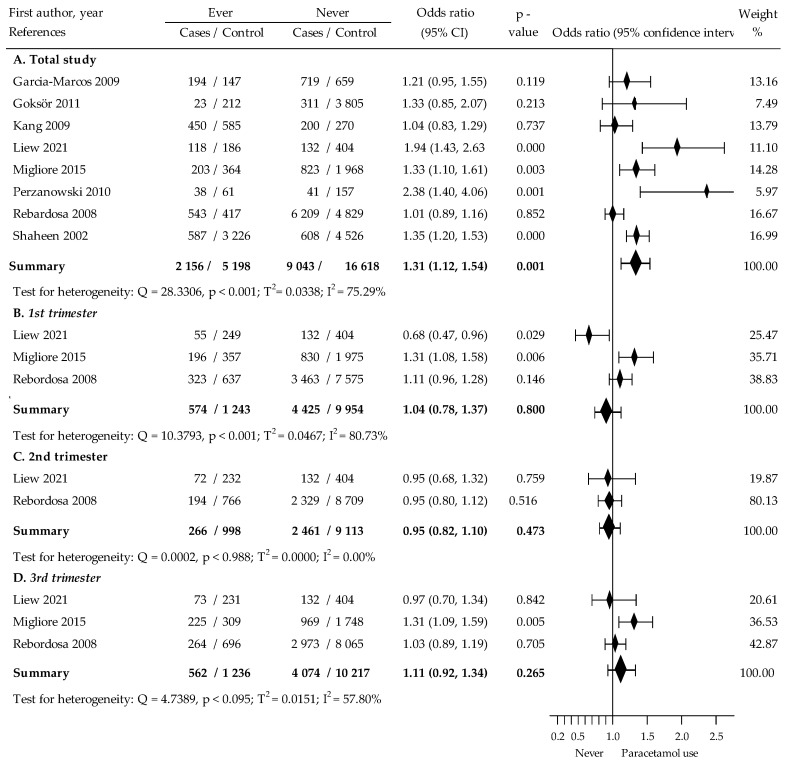
The crude relationship between childhood wheeze and paracetamol use (ever vs. never) during pregnancy and each trimester of pregnancy [22,26,28,29,30,31,32,33].

In the eight studies [22,26,28,29,30,31,32,33] analyzed in order to assess prenatal paracetamol exposure during any time of pregnancy, we noted a significant increased risk of childhood wheeze (crude OR = 1.31, 95% CI: 1.12 to 1.54, *p* < 0.002; with relatively high heterogeneity, I^2^ = 75.29%), (Figure 3). The Begg and Mazumdar’s test for rank correlation indicated no evidence of publication bias (Kendall’s tau b = 0.333, z = 0.939, *p* < 0.349). Egger’s test for regression intercept also demonstrated no evidence of publication bias (b0 = 2.161 (95% CI: −1.775 to 6.097), t = 1.344, *p* < 0.229). 

The use of paracetamol in the first trimester of pregnancy in three studies [22,26,32] indicated a marginal, insignificant increase in the risk of wheezing in childhood (crude OR = 1.04, 95% CI: 0.78 to 1.37, *p* > 0.801, I^2^ = 80.73%), (Figure 3). The results of Begg’s test were inaccessible. Egger’s test did not indicate evidence of publication bias (b0 = −3.819, 95% CI: −56.874 to 49.237, t = −0.915, *p* > 0.529). 

Two studies [22,32] have been identified that meet the inclusion criteria, in assessing the association between paracetamol exposure during the second trimester of pregnancy and childhood wheezing revealed convergent results (OR = 0.95, 95% CI: 0.68 to 1.32, *p* > 0.760 and OR = 0.95, 95% CI: 0.80 to 1.12, *p* > 0.517; respectively), (Figure 3). However, it is difficult to draw reliable result on their basis. 

The crude odds ratio (OR) for the risk of wheezing in children of mothers using paracetamol in the third trimester of pregnancy was 1.11, 95% CI: 0.92 to 1.34, *p* < 0.266, I^2^ = 57.80%, based on three studies [22,26,32], (Figure 2). Egger’s test did not indicate evidence of publication bias (b0 = −0.277, 95% CI: −55.078 to 54.525, t = −0.0641, *p* < 0.959). Results of Begg’s test were inaccessible.

## 4. Discussion

The aim of our systematic review with meta-analysis was to summarize the current evidence on the exposures associated with paracetamol use in utero, focusing on postnatal breathing disorders in children. The study is important for the development of clinical recommendations regarding the consumption of paracetamol during pregnancy. The results of our systematic review and performed meta-analysis indicate a significant increase of the risk of asthma (crude OR = 1.34, 95% CI: 1.22 to 1.48, *p* > 0.001); or wheezing (crude OR = 1.31, 95% CI: 1.12 to 1.54, *p* > 0.002) among children with a history of prenatal exposure to paracetamol. 

Singh et al. [34] noted that the odds ratio for the asthma outcome in the offspring of mothers who used paracetamol in the prenatal period in any trimester of pregnancy was 1.28, 95% CI: 1.13 to 1.39. Fan et al. [35] also held the opinion that prenatal paracetamol exposure was significantly associated with the increased risk of child asthma. In their work, OR = 1.19, 95% CI: 1.12 to 1.27. In turn, Eyers et al. [36] showed increased risk of recurrent wheeze in the children of women who were exposed to paracetamol during pregnancy. In their study, OR was 1.21, 95% CI: 1.24 to 1.44. Paracetamol use during pregnancy can affect both the mother and the fetus. Researches of fetal exposure to paracetamol have concerns on: premature birth [37], neurological development [38] low birth weight [39], hyperactivity disorder/hyperkinetic disorder or adverse development issues [40,41], and other birth defects [42,43]

Several limitations should be identified with regard to our study. Firstly, various prenatal ailments and illnesses may themselves have an impact on the risk of postnatal respiratory disorders. In addition, from the studies included into our meta-analysis, it was not possible to obtain confounding factor data that could have an impact on the final results of our analysis. It is difficult to conclude at what age prenatal paracetamol exposure affects children. Secondly, these are observational studies extended over time. During their duration, we cannot avoid the influence of various factors that may affect the final result. Furthermore, the study drug may have been administered to the children post-partum, as mothers who take paracetamol in pregnancy may be more likely to give paracetamol to their children. There are a number of other methodological problems that are also relevant for the interpretation of the results. Firstly, as a meta-analysis of observational studies, it was prone to the bias (e.g., recall and selection bias) inherent in the original studies. Secondly, most of the studies were observational in nature, did not establish a dose–response relationship, and were conceived to be subject to numerous errors and misleading outcomes regarding period of administration. Indeed, in some studies, a progressive increase in risk associated with increasing number of days of prenatal paracetamol exposure, or increased frequency of use, was observed [29,31,32,33]. Moreover, a limitation may posed by publication high statistical heterogeneity.

## 5. Conclusions

In summary, the results of our study confirmed that maternal paracetamol use in pregnancy is associated with an increased risk of asthma or wheezing in their children. The current findings are consistent with results of previous meta-analyses showing increase in asthma/wheeze symptoms from paracetamol exposure. We believe paracetamol should be used with caution by pregnant women, and at the lowest effective dose, for the shortest duration. Long-term use or the use of high doses should be limited to the indications recommended by a physician, while the mother-to-be should be under constant supervision.

## Figures and Tables

**Table 1:** Characteristics of included studies evaluating the association between prenatal paracetamol intake and asthma or wheezing risk in childhood.

Author, Year Country		Study	Exposure Classification Research Period (Years)	The Children’s Respiratory Disorders	Age of Children’s Diagnosis	Population: Paracetamol Use	Child With Asthma or Wheezing	Outcome Period (Months)	Nos Scale
Studies Included in Meta-Analysis									
1.	Liew [22], 2021 USA	Environment and Pregnancy Outcomes Study Cohort study	Paracetamol use during pregnancy: 1st trimester, 2nd trimester, 3rd trimester, ever. 2003–2007	Asthma: diagnosed by medical professional Wheezing	early childhood	958	Asthma: 118 Wheeze: 304	48	9
2.	Piler [23], 2018 Czech Republic/Brno and Znojmo regions	Czech European Longitudinal Study of Pregnancy and Childhood	Paracetamol use during pregnancy. 1991–1992	Paediatrician-diagnosed asthma	3, 5, 7 and 11 years	1105	Asthma: 41	132	9
3.	Magnus [24], 2016 Norway	Norwegian Mother and Child Cohort Study	Paracetamol use during pregnancy. 1999–2014	Childhood asthma	3 years; 7 years	34,703	Asthma: 1751	36	9
4.	Liu [25], 2016 Denmark	Danish National Birth Cohort	Paracetamol use during pregnancy: 1st trimester, 2nd trimester, 3rd trimester, ever. 1996–2010	Asthma: at least two prescriptions for inhalants or cases diagnosed by a hospital doctor.	3 years or later	63,652	Asthma: 7644	36	8
5.	Migliore [26], 2015 Italy	Nascita e INFanzia: Effeti dell Ambiente study	Paracetamol use during pregnancy: 1^st^ trimester, 3^rd^ trimester. 2005–2013	Asthma: diagnosed by doctor Wheezing or whistling: at least one episode	18 months	3358	Asthma: 185 Wheeze 535	18	7
6.	Andersen [27], 2012 Denmark	Danish Medical Birth Registry	Paracetamol use during pregnancy: 1^st^ trimester, both 2^nd^ and 3^rd^ trimesters, ever. 1996–2008	Asthma: hospital diagnosed, anti-asthmatic drug prescription	median—6.8 years	197,060	Asthma: 24,506	~82	8
7.	Goksör [28], 2011 Sweden	Swedish Medical Birth Register	Paracetamol use during pregnancy. 2003	Asthma: Inhaled corticosteroid-treated Wheezing: three or more episodes	6, 12 months and 4, 5 years	4496	Asthma: 258 Wheeze: 235	54	7
8.	Perzanowski [29], 2010 USA	Columbia Center for Children’s Environmental Health	Paracetamol used during pregnancy by low-income women. 1998–2006	Asthma: self-reported Wheezing: self-reported	5 years	297	Asthma: 99 Wheeze: 99	60	7
9.	Kang [30], 2009 USA	The Yale Study	Paracetamol used in 1st and 3rd trimesters pregnancy. 1997–2000	Asthma: diagnosed by a doctor or health professional	6 years +/− 3 months	1505	Asthma: 172	72	7
10.	Garcia-Marcos [31], 2009 Spain	Murcia (Spain) Study	Paracetamol use during pregnancy.	Wheezing: self-reported	4.08 +/− 0.8 (3–4 years)	1741	Wheeze: 341	36–60	8
11.	Rebordosa [32], 2008 Denmark	Danish National Birth Cohort study	Paracetamol use during pregnancy: 1st trimester, 2nd trimester, 3rd trimester, ever. 1996–2003	Asthma: symptoms reported, physician-diagnosedWheezing: self-reported	18 months—wheeze; 7 years—asthma	12,733	Asthma: 12,530 Wheeze: 11,980	84	9
12.	Saheen [33], 2002 UK	Avon Longitudinal Study of Parents and Children	Paracetamol use during pregnancy. 1992–1999	Wheezing: self-reported	30–42 months	8942	Wheeze: 1195	30–42	9

## Data Availability

The data are available upon request from the corresponding author.
